# The Development of Psychiatric Illness and Chemoprophylaxis of Botulinum Toxin in Migraine: A Narrative Review

**DOI:** 10.7759/cureus.32998

**Published:** 2022-12-27

**Authors:** Ayushi S Dhengare, Darshna G Fulmali

**Affiliations:** 1 Physiology, Jawaharlal Nehru Medical College, Datta Meghe Institute of Medical Sciences, Wardha, IND; 2 Department of Anatomy, Jawaharlal Nehru Medical College, Datta Meghe Institute of Medical Sciences, Wardha, IND

**Keywords:** auditory stimulus, visual stimulus, stress, dementia, depression, bipolar disorder, panic disorder, anxiety disorder, episodic migraine, chronic migraine

## Abstract

A migraine is not just a headache. It is an extremely prevalent neurological condition marked by periodic episodes of unilateral headache, with more than 10 million cases yearly. Migraine often begins at the age of puberty. It substantially impacts the brain and, consequently, psychiatric behavior linked with frequent migraine attacks that may be moderate to severe in intensity. A crucial aspect of migraine variability is comorbidity with other neurological diseases, vascular diseases, and mental illnesses. Psychiatric disorders related to migraine include anxiety disorders, panic disorder, bipolar disorder, depression, etc. It is also estimated that people suffering from migraine are about five times more likely to develop depression than others without migraine. The stimulus for migraine is stress, lack of sleep, skipped meal or fasting, visual stimulation due to high intensity of light, auditory stimulus due to noise, and olfactory stimulus due to a pungent smell. A majority of patients suffer from migraine attacks triggered by noise, some due to visual stimulation, and a few due to perfumes or other odors that trigger their migraine. Diagnosis of this is primarily dependent on history taking and clinical evaluation. Migraine can be classified depending on whether an aura is present or absent. It can further be divided based on the frequency of headaches into episodic migraine or chronic migraine, which may be determined by the duration of the headache. The development of migraine is influenced by both genetics and the environment. It has a detrimental effect on children’s quality of life. A comprehensive analysis of psychiatric illnesses in migraine contributes to early diagnosis and proper treatment of the disease. Also, having a healthy lifestyle (including exercise, a balanced diet, and enough sleep) seems to prevent and improve the condition. Headache in migraine is resistant to medical treatment but responds well to botulinum toxin. This review primarily focuses on the psychiatric issues like depression and anxiety that often accompany migraine. The article also highlights the effects of botulinum toxin on migraine.

## Introduction and background

The word migraine is obtained from the term hemicrania, which is a Greek word that means one-sided headache [1]. Migraine is a frequent neurological headache disorder that is caused due to increase in the excitability of the central nervous system. It is one of the most disabling health issues in the world, and its diagnosis primarily depends on the severity of the headache and its related symptoms. The headache is usually unilateral. The effects of migraine are seen in the workspace, social activities, and relations leading to poor quality of life [2].

The article’s main objective is to study how migraine is frequently associated with psychiatric illnesses such as major depressive disorder, anxiety disorders, and bipolar disorders [3]. Nowadays, migraine along with psychiatric disorders is becoming challenging for the healthcare management system all around the world. Studies have put forward that people experiencing migraine, precisely patients with chronic migraine (CM), are at a high probability of developing anxiety, depression, and suicidal behavior in contrast to the rest without migraine [4].

## Review

Migraine has now become a familiar term as the number of people suffering from this keeps on increasing every year at a high pace. It is a frequent and incapacitating major headache disorder. On the grounds of duration, it is classified into two broad categories-episodic migraine and CM. Episodic migraine is described as a condition in which headache is less frequent than 15 days per month. Whereas, if the headache frequency is more or equal to 15 days a month along with the characteristic of migraine then it is known as CM [5]. Numerous population-based investigations have found a link between migraine and mental illness. It is also proven that individuals with prolonged migraine are more likely to experience psychiatric comorbidity than those with episodic migraine [6]. The research findings are consistent and demonstrate a higher chance of developing depression, bipolar disorders, a broad range of anxiety disorders, and post-traumatic stress disorder [7].

Migraine and psychiatric illness: shared pathophysiology

Due to the same clinical characteristic (like headache and mental disturbances) of both migraines and mental disorders such as depression, anxiety, mood swings, and bipolar disorder, they are inextricably linked. People with migraine are more prone to anxiety disorders than the general healthy population [8]. Psychiatric illnesses and migraine have a bidirectional relation, with one condition raising the risk of others [9]. Migraine is mainly accompanied by psychiatric comorbidity. Comorbidity is indicated by the existence of any other coexisting ailment in patients with a specific core disease [10]. Clinical and epidemiological investigations have both provided a correlation between this disease and psychological issues. The anxiety disorder clinic sample seemed to have a 67% probability of migraine [11].

Apart from episodic migraine and CM, migraine has two more types that are migraine with aura and migraine without aura. A migraine with aura is an intense headache accompanied by other symptoms such as light sensitivity, crooked vision, buzzing in the ears, or dizziness. Around 90% of victims reported a visual aura, making it the most prevalent manifestation. Aura is a warning signal that manifests in your vision and other senses to let you know that headache is about to begin. In migraine without aura, there is no such sign before migraine, but this may include other features like nausea and vomiting [12]. Anxiety, depression, and fatigue may accompany migraines without auras in certain situations. These symptoms usually appear some hours before headache onset. In the absence of an aura, some people who suffer from this form of migraine may experience additional symptoms, such as feeling thirsty or exhausted or seeking sweets. According to the American Headache Society (AHS), migraine without aura can continue for up to 72 hours. Aura-accompanied migraines are linked to the development of ischemic stroke [13]. Some data for different forms of migraine are given in Table 1. Table 1 depicts the number of people affected by a particular type of migraine [14].

**Table 1:** Forms of migraine and their percentage [14]

Types of migraine	Cases per 100 people	Proportion as percentage
Migraine with aura	34	39.5%
Migraine without aura	24	27.9%
Migraine with aura + without aura	10	11.6%
Probable migraine with aura	6	7.0%
Probable migraine without aura	9	10.5%
Migraine without aura + probable migraine with aura	2	2.3%
Migraine without aura + menstrual migraine	1	1.2%

Features of migraine and psychiatric disorder

The clinical features of migraine are generally observed as episodic headaches marked by pain felt on one side of the head (known as unilateral headaches). The intensity of pain may be moderate to severe in intensity [15]. Multiple common clinical characteristics between migraine and psychiatric disorders may make it more difficult or take longer to diagnose the emergence of psychiatric disorders in migraine sufferers [16]. Amongst the patients of migraine, especially those with CM, have more probability of undergoing extreme anxiety and suicidal tendencies [17]. A significant co-occurring condition for people with bipolar disorder is migraine. In addition to aggravating the root causes of bipolar illness, it also increases the risk of depressive episodes.

Along with this, patients with bipolar disorder also pose a risk of acquiring comorbid migraine. Therefore, research has shown that migraine and psychiatric disorders have a bidirectional relationship [18]. The correlation theory between migraine and psychological disorders originates with anxiety in youth and adolescence, followed by migraine and later depression. As in the case of migraine, psychiatric disorders have also been studied to run in the family. Comorbidity amid migraine and psychiatric disorders has been significantly studied; still, the principle behind this occurrence remains very unclear, but much research related to this is still under progress [19].

Both migraines and depression exhibit bidirectional relations, with one increasing the risk of developing the other. In most cases, depression develops after the beginning of a major headache disorder, therefore this bidirectional association appears to be specific to migraine. Studies point towards a two-fold increase between migraine and depression in siblings and twins [20]. Countless surveys and research are directed towards the decline in the standard of living of the patients, which leads to limiting themselves from daily activities, job, and school absenteeism, becoming dependent on others for personal work, and seeking more health services [21]. Migraine attacks may negatively affect the family, social life, job efficiency, and output of the patient with migraine [22]. Along with psychiatric disorders, migraine also increases the chance of rheumatoid arthritis [23].

Diagnosis

There is no such precise test for the diagnosis of migraine. Therefore, to reach an appropriate diagnosis, a clinician looks at the patterns of recurrent headaches and related symptoms [24]. While evaluating a patient, it is essential to note that headache is just an indication and not a diagnosis [25]. The clinicians also look for family history, if any. According to the studies, genetics and heredity are also crucial factors influencing a person’s vulnerability to migraine [26]. Significant signs of migraine may include throbbing headache, nausea, vomiting, sensitivity to light, and high sound intensity (photophobia and phonophobia, respectively) [27]. A survey has been done and studied on Cureus to observe the parameters present in migraine sufferers. The parameter and results of the study are as follows in Table 2 [28].

**Table 2:** The parameter and results of the study [28]

Parameter	Results
Nausea	160 (57.76%)
Vomiting	115 (41.52%)
Aura	44 (15.88%)
Photophobia/phonophobia	161 (58.12%)
Neck pain	109 (39.35%)
Alcohol addict	33 (8.2%)
Smoking	21 (5.2%)
Consumption of Pan or betel	83 (20.7%)

Botulinum toxin and its use in migraine

Both botulinum toxin A and botulinum toxin B have been shown to be effective in treating neuropathic pain. Botulinum toxin is also referred to as Botox or BoNT. However, BoNT-A is more extensively utilized due to its least noticeable side effect and prolonged durability of treatment [29]. Botulinum toxin type A has been used for more than 15 years now for the management of CM headaches. Also, it has recently emerged as a safe alternative for preventing persistent migraine. Upcoming research is progressively highlighting its migraine preventive mode of action [30]. Botulinum toxin is a neurotoxin that is produced by the bacteria Clostridium botulinum. Botulinum toxin is generally used in the form of injections. It is well tolerable and has few or relatively minimum side effects. It is also known as “miracle poison” [31]. A study performed on a population published on PubMed stated that within a period of 8 to 12 weeks, post administering the injection of botulinum toxin, there was a 57% reduction in consumption of medicine in case of acute headache [32]. Botulinum toxin A is also referred to as BoNT/A. It not only helps in migraine but is also beneficial for addressing a broad range of involuntary muscular contraction diseases such as cervical dystonia, hemifacial spasm, blepharospasm, etc. upcoming clinical pieces of evidence indicate BoNT/A can reduce pain related to migraine and other headaches forms [33].

Botulinum toxin type A injections are now primarily administered to individuals who undergo chronic headaches daily, with a migraine element in their clinical presentation [34]. In people suffering from episodic and CMs, botulinum toxin has been proven potent in diminishing the prevalence of headaches, severity, and impairments due to headaches [35]. The neurotoxin BoNT/A suppresses pain sensitization via peripheral and perhaps central mechanisms by interfering with neuropeptide release and receptor translocation associated with trigeminal nociception. Still, a large number of researches are yet to be done to realize the medicinal potential of the toxin ultimately and to be aware of its mechanism of action [36].

The US Food and Drug Administration authorized botulinum toxin as a prophylactic treatment for CM in 2010. Research on the toxin also suggests that this toxin is independent of the time span of the therapy and the site of the headache [37]. The maximum number of subjects who obtained botulinum toxin for CM observed benefits in their signs and symptoms and general quality of life [38]. For individuals with persistent pain issues, botulinum toxin provides an exclusively advanced alternative and approach. Specifically, those people with migraines frequently experience intense exhaustion, nausea, impaired attention, lack of appetite, gain in body weight, and hair loss as an out-turn of the negative impacts of the medication consumed for the treatment. Such adverse reactions have not been reported by the patients in response to the use of botulinum toxin [39].

Botulinum toxin A has a well-established track record of success in treating patients with persistent migraines. Yet there are several unresolved issues, thus making it a matter to look after. Due to the rareness of the disorder, there is no RCT-proven preventive medication for hemiplegic migraine, an odd form of migraine with aura. In two research studies and 11 patient clinical trials, the usage of botulinum toxin for hemiplegic migraine has been documented. Nine of the 11 patients in the case trial reported a decline in the regularity of prolonged migraine aura [40]. Botulinum toxin type A, 25 U, an injection administered in the pericranial route, has been demonstrated to be a feasible therapy that greatly lowers intense drug use, vomiting related to migraines, and recurrence and extremity of the condition. Botox injections type A typically minimize the discomfort brought on by the diseases [41]. Although this approach will cost the patients money, it will relieve their symptoms far more effectively than those of other treatments now available. The administration of BoNT does not raise the chance of abnormalities in pregnant women or fetuses. Yet, using BoNT to manage illness during gestation needs patients to provide fully informed permission [42].

Mechanism of action of botulinum toxin

Botulinum toxin type A is a beneficial and reliable alternate pain medication that provides a great advantage to people unable to react to opioid therapy. Botulinum toxin has an extended term of effect, extending up to 22 weeks from the first therapy, making it a pretty promising therapeutic option for people suffering from chronic pain. The function of botulinum toxin is to paralyze muscles and lower pain responses by preventing the production of acetylcholine from nerve terminals [43]. An isolated protein called botulinum toxin type A restricts muscular contractions by suppressing the secretion of acetylcholine via presynaptic neurons. Botulinum toxin also inhibits the production of nociceptive mediators such as substance P, glutamate, and calcitonin gene-related peptide, according to preclinical in vitro and in vivo research, which suggests that BoNT-A lessens nociceptive input into the nervous system from the periphery [44]. Botox further inhibits peripheral autonomic fibers and involuntary smooth muscle as well as glands having ducts. The immediate impact of Botox is not evident on the central nervous system (CNS) because botulinum toxin does not traverse the blood-brain barrier. Therefore it is disabled during retrograde axonal transportation [45]. In the European Union (EU) and North America, the sole medication exclusively certified for the prophylaxis of headaches in individuals with CM is an intramuscular injection of botulinum toxin serotype A [46].

In contrast to the placebo, Botox treatment contributed to numerically remarkable and clinically appropriate gains in productivity and health-related quality of life. The usage of BoNT-A is a low-cost alternative for the management of several forms of migraines, which would include chronic, episodic, unilateral, and vestibular migraines. BoNT-A could lower the number of migraine incidents every month and the level of discomfort caused due to the high intensity of headaches and associated symptoms daily. The minimum, as well as the maximum age criteria for administration of BoNT-A or to undergo botulinum toxin therapy, is 12 years to 86 years of age. Although the doses of Botox prescribed differ from physician to physician and from patient to patient, most studies prefer 155 units of botulinum toxin [47].

Researches also demonstrate that botulinum toxin A might significantly relieve depression symptoms and is a reliable supplementary therapeutic for individuals undergoing antidepressant medication [48]. The conceptual basis of BoNT is not just limited to depression but applies to any condition linked with a surplus of unfavorable emotions. As a result, the usage of BoNT has a transdiagnostic ability that is largely validated by the suitable clinical study on borderline personality syndrome (BPS) and social anxiety syndrome. BoNT treatment incorporates standard treatment modalities such as pharmaceutical, relaxation activities, behavioral therapy, and socialization therapy [49]. The freshly conducted research has revealed that botulinum toxin injections in the glabellar frown lines reduce anxiety and depression symptoms. Additional medical trials with more extensive population samples are desired to demonstrate the benefit and security of botulinum toxin injectables utilized to address psychiatric illnesses [50].

Management

The significance of healthy lifestyle changes is getting progressively essential in the case of migraine. Physical fitness, exercising regularly, a balanced meal, and an improved way of living, such as quality sleep and drug abstinence, each greatly benefit to reduce attack occurrence and intensity. These lifestyle modifications must be considered while developing migraine control techniques [51]. Unsatisfactory sleep, whether in terms of quality or quantity, has been linked to an increased incidence of migraine episodes in migraineurs. Therefore controlling sleep disruptions appear essential in this headache condition [52]. Medical and community research has found a strong link between regular workouts and migraine. The latest random controlled investigations focusing on the efficacy of aerobic exercise as a migraine prevention therapy have found that it significantly improves the clinical manifestation of migraine and associated discomfort, causing great relief from regular migraine attacks [53].

The occurrence of depressive episodes is an invalidating prognostic marker for cognitive behavioral therapy in headaches. It has been observed that patients undergoing this treatment benefit and show a substantial rate of recovery than those patients who do not opt for such therapies. Therefore, the minimal effect is seen on their discomforts such as headache, anxiety, depression, and quality of life. It was also recorded that in an interval of 4 months post-treatment, the results of improvement were well maintained. Thus, this upcoming comprehensive cognitive behavioral therapy treatment seems influential, trustworthy, and deserving of future research studies [54].

## Conclusions

Migraine is a pervasive and incapacitating disorder that is often connected with a vast spectrum of psychiatric comorbidities, including anxiety, depression, panic, bipolar, and sleep disorders. Concerning the findings in the review, the correlation between psychiatric issues and migraine are complex, with a bidirectional connection between the two. The recent advancement in treating migraine and the associated neuropathic pain with botulinum toxin has come up, benefiting a large population of sufferers. Still, the full potential of this toxin is undiscovered and needs more research.
